# Modeling and Optimization of Bidirectional Clamping Forces in Drilling of Stacked Aluminum Alloy Plates

**DOI:** 10.3390/ma13122866

**Published:** 2020-06-26

**Authors:** Jintong Liu, Anan Zhao, Piao Wan, Huiyue Dong, Yunbo Bi

**Affiliations:** 1 State Key Laboratory of Fluid Power and Mechatronic System, College of Mechanical Engineering, Zhejiang University, Hangzhou 310027, China; liujintong@zju.edu.cn; 2 Key Laboratory of Advanced Manufacturing Technology of Zhejiang Province, College of Mechanical Engineering, Zhejiang University, Hangzhou 310027, China; 21625195@zju.edu.cn (P.W.); donghuiyue@zju.edu.cn (H.D.); 3 Aviation Industry Corporation of China Xi’an Aircraft Industry (Group) Limited Company, Xi’an 710089, China; zhaoaa@avic.com

**Keywords:** drilling, stacked aluminum plates, interlayer gap, bidirectional clamping force

## Abstract

Interlayer burrs formation during drilling of stacked plates is a common problem in the field of aircraft assembly. Burrs elimination requires extra deburring operations which is time-consuming and costly. An effective way to inhibit interlayer burrs is to reduce the interlayer gap by preloading clamping force. In this paper, based on the theory of plates and shells, a mathematical model of interlayer gap with bidirectional clamping forces was established. The relationship between the upper and lower clamping forces was investigated when the interlayer gap reaches zero. The optimization of the bidirectional clamping forces was performed to reduce the degree and non-uniformity of the deflections of the stacked plates. Then, the finite element simulation was conducted to verify the mathematical model. Finally, drilling experiments were carried out on 2024-T3 aluminum alloy stacked plates based on the dual-machine-based automatic drilling and riveting system. The experimental results show that the optimized bidirectional clamping forces can significantly reduce the burr heights. The work in this paper enables us to understand the effect of bidirectional clamping forces on the interlayer gap and paves the way for the practical application.

## 1. Introduction

Under the premise of satisfying the structural strength requirements, a primary pursuit for modern aircraft is to remain lightweight. Therefore, the usage of lightweight materials such as titanium alloy, aluminum alloy and composite materials is rapidly increasing [1,2,3]. In aircraft assembly, the connection quality of lightweight stacked structures directly affects the fatigue performance and service life of the aircraft. To improve the drilling and connection quality of aircraft components, many automatic drilling and riveting systems are introduced into aircraft manufacturing industries [4,5,6]. During the automatic drilling process of stacked metal plates, one of the most outstanding issues is the formation of interlayer burr [7,8]. The existence of interlayer burr not only affects the hole quality but also leads to connection decline or even failure [9]. Moreover, after completing the drilling process, the deburring operation has to be performed to eliminate the interlayer burrs. The stacked plates need to be disassembled and reassembled several times. These extra operations are not only time-consuming but also affect the assembly precision [10,11].

Many investigations have been conducted on the burr formation and elimination in drilling metal materials. Researchers indicate that the process parameters, workpiece parameters and tool parameters are the most significant influencing factors of burr formation [12,13,14]. Abdelhafeez et al. [15] conducted fatigue tests on as-drilled and duburred specimens made of titanium and aluminium alloys and they found that the deburring dramatically increased the fatigue performance of the Ti–6Al–4V and AA7010 samples. Hu et al. [16] provided an analytical model to investigate the deflections of top and bottom metal sheets under complex drilling conditions, and the interfacial burr height can be predicted with the proposed model. Abdelhafeez Hassan et al. [17] proposed an analytical model to predict entrance burr dimensions for ductile metals and extended it to account for interlayer burrs in CFRP-metallic stacks. The predicted sizes of interlayer burrs were accurate to within 20% of the experimental measured results. To enhance tool performance, Rodríguez-Barrero et.al. [18] and Fernández-Abia et al. [19] tested several coating materials for tools and compared their performance. The results are helpful toward the selection of tools and the optimization of tool parameters in stack drilling. Besides, the interlayer gap between adjacent layers also contributes a lot to the formation of interlayer burrs [20]. The main reason for the interlayer gap formation is that the elastic bending of the upper plate and lower plate occurs to different degrees due to different force conditions. The interlayer gap reaches its maximum value when the upper plate is drilled through and the lower plate is being drilled [21]. Bu et al. [22] presented an analytical model of the interlayer gap formation to predict the interlayer burr height. They conducted drilling experiments to understand the difference between the interlayer burr height and the interlayer gap. Gao et al. [23] studied the interlayer gap formation and non-coaxiality occurrences in the drilling of stacked structures of broad skins and narrow stringers. They found that stack stiffness, drilling force and pressing force are the significant influencing factors.

Many methods of controlling the interlayer gap and eliminating burrs have been studied, of which preloading pressing force is considered as the most common and effective way [24]. Choi et al. [25] proposed a finite element model of interlayer gap formation for multi-layered materials and investigated the influence of pressing location on gap size. Liang et al. [26] discussed the effect of the thrust force and pressing force on both interlayer gaps and non-coaxiality for CFRP/Al stack drilling, and found that the interlayer gap increased with the increase in pressing force but decreased with the pressing force. Lei et al. [27] presented a theoretical relationship between the clamping force and the interlayer burr formation and introduced the shell theory to calculate the optimal pressing force. However, most of the existing studies on the preloading pressing force are based on the one-sided clamping method.

**Figure 1:**
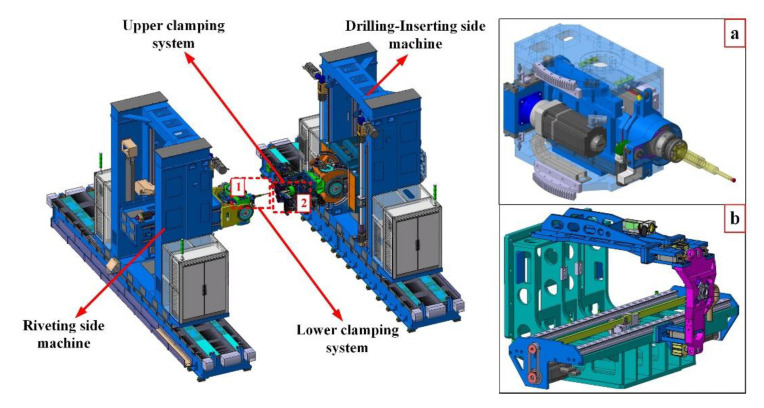
The dual-machine-based automatic drilling and riveting system with bidirectional clamping feet: (**a**) lower clamping system; (**b**) upper clamping system.

As shown in Figure 1, the dual-machine-based automatic drilling and riveting system is designed for the drilling and riveting of aircraft panels. The system adopts a horizontal layout and consists of two machine tools applied for drilling-inserting and riveting, respectively. The drilling and riveting system employs a bidirectional clamping way to reduce the interlayer gap and eliminate interlayer burrs. Besides, the diameter of the lower presser foot is smaller than the upper presser foot to the internal structure of the panels. There are many differences between the bidirectional clamping way and the traditional one-sided clamping, so the previous research results based on one-sided clamping do not apply to the dual-machine-based riveting system. During the drilling process in practical engineering, the bidirectional clamping forces are usually determined by manual experience due to the lack of theoretical guidance, which would lead to hole errors and affect the connection quality.

Therefore, this paper is greatly concerned with the effect of bidirectional clamping forces on the interlayer gap, which has received little attention in previous investigations on stack drilling. We aim to find the optimal clamping forces that reduce both the interlayer burrs and the deflections of stacked plates. The remainder of the paper is organized as follows: In Section 2, a mathematical model of the interlayer gap is developed, the relationship between the upper and lower clamping forces is studied and the optimization is performed. Section 3 verifies the mathematical model through numerical simulation, with the details of the finite element modeling (FEM) provided. In Section 4, drilling experiments of stacked aluminum alloy plates are conducted with the automatic drilling and riveting system. The results and discussion are presented. Lastly, the conclusion and future works are summarized in Section 5.

## 2. Analytical Modeling and Optimization of Bidirectional Clamping Force

### 2.1. Modeling of the Interlayer Gap with Bidirectional Clamping Forces

**Figure 2:**
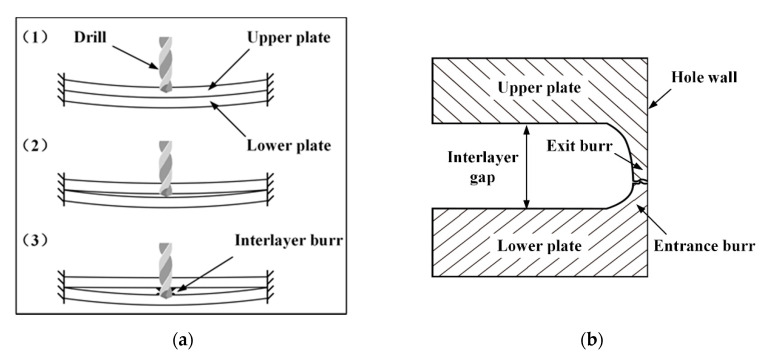
The interlayer burr formation during drilling of stacked aluminum plates: (**a**) the three stages of the drilling process; (**b**) the interlayer burr situation in stage 3.

In order to eliminate the interlayer burr by controlling the interlayer gap, the modeling of the interlayer gap in stack drilling is performed. As shown in Figure 2a, the drilling process of stacked aluminum plates can be decomposed into three stages. In stage 1, the upper plate is drilled and undergoes the drilling thrust force while the lower plate bends downward due to the pressure transmitted from the upper plate. In this stage, the initial interlayer gap is eliminated. In stage 2, the upper plate is drilled through and the drill bit reaches the lower plate, then spring-back occurs on the upper plate while the deflection of the lower plate increases due to the drilling thrust force. The interlayer gap begins to form and increase in stage 2. In stage 3, the drill bit is fully contacted with the lower plate. The drilling thrust force is exclusively applied on the lower plate while the upper plate endures a negligible force. The different force conditions of the upper and lower plates result in the maximum interlayer gap during the whole drilling process. The interlayer burr situation in stage 3 is shown in Figure 2b. The interlayer gap includes the upper plate exit burr and lower plate entrance burr, and both are strictly related to the interlayer gap formation, so diminishing the interlayer gap could be an efficient way to eliminate the interlayer burr.

**Figure 3:**
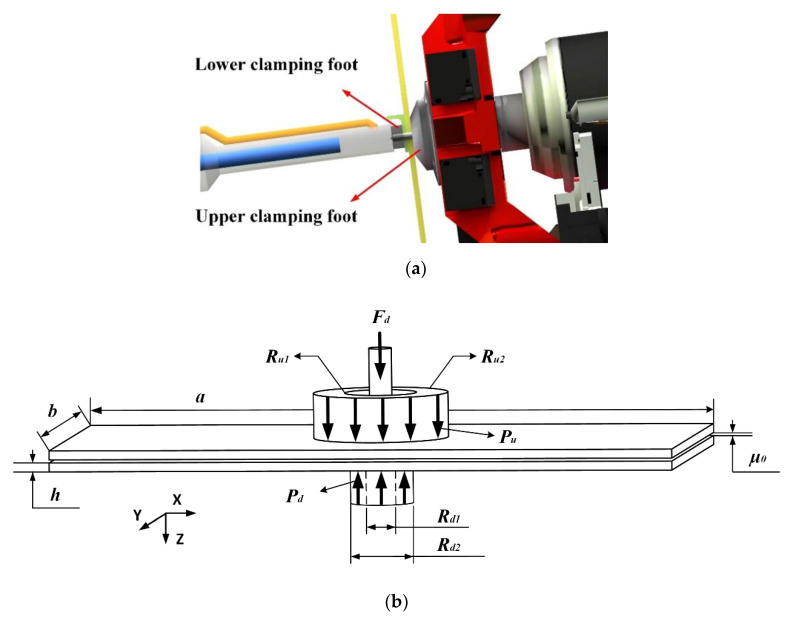
(**a**) The bidirectional presser feet of the automatic drilling and riveting system; (**b**) the schematic of stack drilling with bidirectional presser feet.

In the automatic drilling and riveting system, as shown in Figure 3a, the bidirectional presser feet are utilized to apply clamping forces on both sides of the drilling area. The schematic of the stack drilling with bidirectional clamping forces is demonstrated in Figure 3b, where: $F_{d}$ represents the drilling thrust force; $P_{u}$ and $P_{d}$ represent the pressures applied on the clamping areas of the upper side and lower side, respectively; $R_{u1}$, $R_{u2}$, $R_{d1}$, and $R_{d2}$ represent the inner radius and outer radius of the upper presser foot and lower presser foot, respectively; a, b and h are the length, width and height of the plate, respectively; and $\mu_{0}$ is the initial interlayer gap.

**Figure 4:**
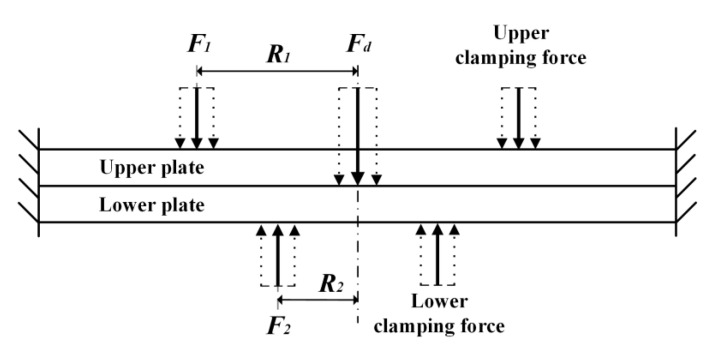
The simplified model of stack drilling with bidirectional clamping forces.

According to the theory of plates and shells [28], a simplified model of the stack drilling with bidirectional clamping forces is established and displayed in Figure 4. The model mainly consists of two stacked rectangular plates with drilling thrust force and bidirectional axially symmetric clamping forces distribution. The thrust force is considered as a concentrated force at the drilling center, while the bidirectional clamping forces in the ring-shaped area are reasonably simplified into circular equivalent forces [29]. The $F_{1}$ substitutes the upper clamping force, which uniformly distributed at a circle with radius $R_{u}$ around the drilling center. Similarly, the $F_{2}$ substitutes the lower clamping force applied at a circle with radius $R_{d}$. The calculation formulas of the equivalent clamping forces are as follows:

(1) $$F_{1}={\pi R}_{u}^{2}P_{u},\;\mathrm{where}\;R_{u}=\frac{R_{u1}+R_{u2}}{2}\;,$$

(2) $$F_{2}={\pi R}_{d}^{2}P_{d},\;\mathrm{where}\;R_{d}=\frac{R_{d1}+R_{d2}}{2}\;,$$

Furthermore, some critical hypotheses are given to ensure the validity of the mathematical model [18]:

1. The stacked plates are both thin plates, with the length and thickness ratio larger than 0.5;
2. Only elastic deformation and small deflection occur to the plates, the maximum deflection does not exceed 1/5 of plate thickness.
3. The edges of the plates are considered as built-in or fixed.

During the drilling process, the interlayer gap has a positive correlation with the interlayer burr size, and the largest interlayer gap occurs at the drilling center [12]. It is obvious that the interlayer gap decreases as the clamping force increases until the two plates are entirely in contact with each other. Therefore, when the interlayer gap at the drilling center is reduced to zero, the clamping forces can be considered to be optimal. With the simplified stack drilling model, we can calculate the total deflection of each plate by superposing the deflection under each load. So, the relationship between the interlayer gap at the drilling center and the bidirectional clamping forces can be established. Then the optimal clamping forces can be obtained when the interlayer gap equals to zero.

To achieve the above objective, firstly, we need to calculate the deflection of each plate under each load. We assume that ω(O→r) represents the deflection at the circle of radius r (r∈[0,b]) caused by a unit force applied at position O. It should be noticed that O could be a point or a uniformly distributed circle. For the upper plate, it only bears the upper clamping force $F_{1}$, so the deflection at the circle of radius r can be represented as Equation (3):

(3) $$\omega_{1}\left(r\right)=F_{1}\cdot \omega \left(R_{u}\to r\right),$$

The lower plate is subject to both drilling thrust force and lower clamping force. The deflection of the lower plate caused by the thrust force $F_{d}$ is determined as Equation (4):

(4) $$\omega_{2d}\left(r\right)=F_{d}\cdot \omega \left(O\to r\right),$$

Similarly, the deflection of the lower plate caused by the lower clamping force $F_{2}$ can be represented as Equation (5):

(5) $$\omega_{2c}\left(r\right)=F_{2}\cdot \omega \left(R_{d}\to r\right),$$

Then, the interlayer gap ∆ω(r) at the circle of radius r around the drilling center O can be calculated with Equation (6):

(6) $$∆\omega \left(r\right)=\mu_{0}-\omega_{1}\left(r\right)+\omega_{2d}\left(r\right)-\omega_{2c}\left(r\right),$$

where $\mu_{0}$ is the initial interlayer gap.

After the drilling process parameters and workpiece parameters are determined, the interlayer gap will become a function of three variables $∆\omega \left(r\right)=\;f\left(F_{1},{\;F}_{2},\;r\right)$, can be expressed as zero, which means ∆ω(r)=0 when r=0. Then, the Equation (6) would only have two variables $F_{1}$ and $F_{2}$. In other words, the relationship between the upper clamping force and lower clamping force when the interlayer gap is zero can be obtained. However, on the premise that the interlayer gap is zero, we still need to find the optimal group of the bidirectional clamping forces. Because different groups of the bidirectional clamping forces will give rise to different deflections of the stacked plates, which will result in the non-coaxiality of the holes [23]. Therefore, the optimization objective of the bidirectional clamping forces is proposed, which is to reduce the degree and non-uniformity of the deflections of the stacked plates.

According to Equation (6), when the interlayer gap equals to zero at the drilling center, the deflections of the upper plate $\omega_{u}\left(r\right)$ and lower plate $\omega_{d}\left(r\right)$ can be expressed as Equations (7) and (8), respectively:

(7) $$\omega_{u}\left(r\right)=\omega_{1}\left(r\right)=\mu_{0}+\omega_{2d}\left(r\right)-\omega_{2c}\left(r\right),$$

(8) $$\omega_{d}\left(r\right)=\omega_{2d}\left(r\right)-\omega_{2c}\left(r\right),$$

If one-sided clamping is adopted, which means $F_{2}=0$, then $\omega_{2c}\left(r\right)$ would be zero. As can be seen from Equation (7) and Equation (8), the deflections of the upper and lower plates $\omega_{u}\left(r\right)$ and $\omega_{d}\left(r\right)$ will both increase. The main reason is that when one-sided clamping is used, the downward bending of the lower plate is increased due to the lack of lower clamping force. As a result, the upper plate has to bend more to ensure that the interlayer gap remains zero. The increase of the deflections of the stacked plates will affect the coaxiality of the hole and ultimately reduce the fatigue performance of the joint. Therefore, compared with one-sided clamping, the bidirectional clamping way is more capable because it can reduce not only the interlayer burr but also the deformation of the stacked plates.

Then, the specific calculation method of the deflection of the thin plate is presented. According to the theory of plates and shells [28], the differential equation of the thin plate bending is shown in Equation (9):

(9) $$\{\begin{array}{c} \frac{\partial^{4}\omega }{\partial x^{4}}+2\frac{\partial^{4}\omega }{\partial x^{2}\partial y^{2}}+\frac{\partial^{4}\omega }{\partial y^{4}}=\frac{q\left(x,y\right)}{D}\\ D=Eh^{3}/12\left(1-v^{2}\right) \end{array},$$

where ω is the deflection of the plate, q(x,y) is the force applied on the plate, (x,y) is a point in the plane coordinate, E is elastic modulus, h is the plate thickness and v is Poisson’s ratio.

In the case of the rectangular plates are constrained on four edges, the boundary conditions can be expressed as Equation (10):

(10) $$\{\begin{array}{c} x=0,x=a\to \omega =0,\frac{\partial^{2}\omega }{\partial x^{2}}=0\\ y=0,y=b\to \omega =0,\frac{\partial^{2}\omega }{\partial y^{2}}=0 \end{array},$$

where the parameter ω can be expressed in the form of double trigonometric series, the detailed calculation process can be referred to our previous job [27].

Then, the Naiver solution of the deflection of the rectangular thin plate can be represented as Equation (11).

(11) $$\omega =\frac{4}{\pi^{4}\mathrm{abD}}\sum_{m=1}^{\infty }\sum_{n=1}^{\infty }\frac{\int_{0}^{a}\int_{0}^{b}q\left(x,y\right)\sin\frac{m\pi x}{a}\sin\frac{n\pi y}{b}\mathrm{dxdy}}{{\left(\frac{m^{2}}{a^{2}}+\frac{n^{2}}{b^{2}}\right)}^{2}}\sin\frac{m\pi x}{a}\sin\frac{n\pi y}{b},$$

Define $P_{0}\left(x_{0},y_{0}\right)$ as the position of the drilling thrust force applied at the lower plate, and $P_{1}\left(x_{1},y_{1}\right)$, $P_{2}\left(x_{2},y_{2}\right)$ are the coordinates of the upper and lower clamping forces applied at the plates, respectively. Their positional relationship can be represented as Equations (12) and (13):

(12) $$\{\begin{array}{c} x_{1}=x_{0}+R_{u}\cos\theta \\ y_{1}=y_{0}+R_{u}\sin\theta \end{array},$$

(13) $$\{\begin{array}{c} x_{2}=x_{0}+R_{d}\cos\theta \\ y_{2}=y_{0}+R_{d}\sin\theta \end{array},$$

With Equations (11)–(13), we can calculate the deflection at any point P(x,y) of each plate under each load in a uniformed coordinate system. Eventually, the deflection caused by unit force at drilling center and clamping areas can be obtained as follows:

(14) $$\{\begin{array}{c} \omega \left(O\to r\right)=\omega_{2d}\left(r\right)/F_{d}\\ \omega \left(R_{u}\to r\right)=\omega_{1}\left(r\right)/F_{1}\\ \omega \left(R_{d}\to r\right)=\omega_{2}\left(r\right)/F_{2} \end{array},$$

### 2.2. Optimization of Two-Side Clamping Force

Based on the above mathematical model of the interlayer gap, the optimization of the bidirectional clamping forces for the automatic drilling and riveting system can be performed. Firstly, we need to acquire the relationship formula between the upper and lower clamping forces when the interlayer gap is zero.

The 2024-T3 aluminum alloy is widely used in airplane structures such as fuselage and wings because of its high strength to weight ratio and excellent fatigue properties [21]. In this investigation, the 2024-T3 aluminum alloy plates are used for stack drilling. The materials of the presser foot and the drill are stainless steel and cemented carbide, respectively. Their material properties are shown in Table 1.

The initial interlayer gap $\mu_{0}$ is set to 0.35 mm and the drilling thrust force $F_{d}$ is set to 130 N according to the actual manufacturing condition [24]. The geometric dimensions of the plates and the presser feet of the automatic drilling and riveting system are shown in Table 2.

Substituting the parameters in Table 1 and Table 2 into Equation (14), the calculation results of the deflections caused by the unit force applied at the drilling center and clamping areas can be obtained. Due to the low convergence rate of the double trigonometric series, when calculating the Naiver solution, the first 20 items of the series are taken as an approximate solution. The calculation results are shown in Table 3.

When the interlayer gap is zero (∆ω(r)=0), substitute the above calculation results into Equation (6), the relationship between the bidirectional clamping forces can be obtained as follows:

(15) $$F_{1}+1.068F_{2}=268.3\left(\mathrm{unit}:N\right),$$

where $F_{1}\geq 0$ and $F_{2}\geq 0$.

Theoretically, when the bidirectional clamping forces satisfy the Equation (15), the interlayer gap would be zero, and the interlayer burr could be eliminated. However, there is an ocean of groups of the bidirectional clamping forces that meet Equation (15), and if not correctly selected, it may lead to excessive and non-uniform deformations of the stacked plates. Therefore, it is necessary to find the optimal group of the bidirectional clamping forces to reduce the interlayer burr and total deflections of the stacked plates.

In order to quantitatively evaluate the uniformity of the deflections of the two stacked plates, an evaluation index T is defined in Equation (16), it represents the average deviation of each plate from the position where the total deformation of the stacked plates is the minimum. The smaller the deflection index T is, the smaller the total deformation of the stacked plates.

(16) $$T=\frac{\left|\omega_{u}-\frac{\mu_{0}}{2}\right|+\left|\omega_{d}+\frac{\mu_{0}}{2}\right|}{2},$$

**Figure 5:**
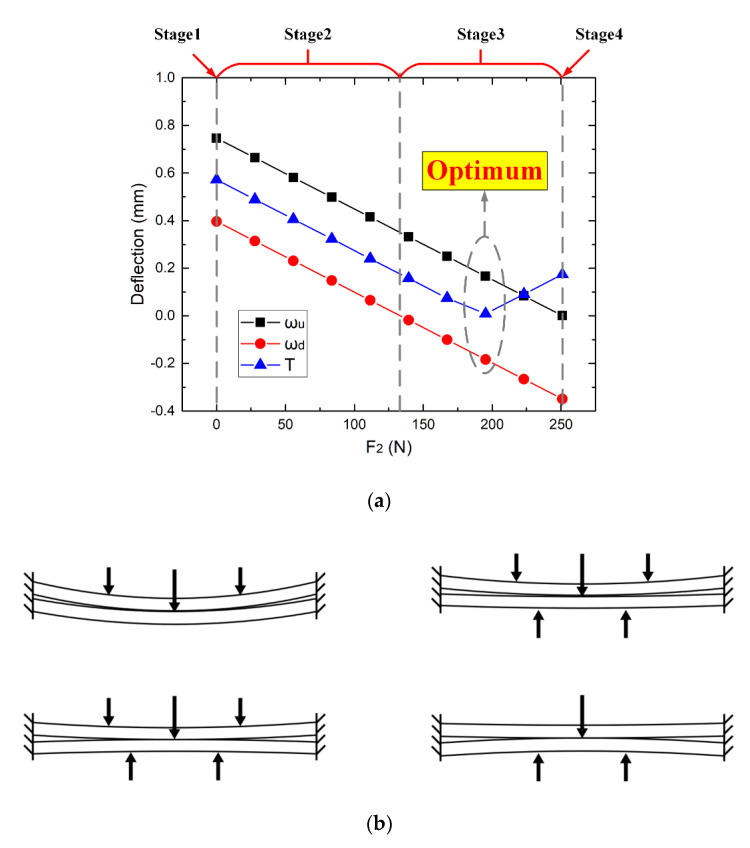
(**a**) The deflections of the upper and lower plate with different bidirectional clamping forces satisfying Equation (15); (**b**) the four stages of the deflections of the stacked plates with the increase of $F_{2}$.

According to Equation (7) and Equation (8), on the premise that the interlayer gap is zero, the deflections of the upper and lower plate with different bidirectional clamping forces can be obtained, with the results represented in Figure 5. The X-axis indicates that the lower clamping force $F_{2}$ increases from zero while the corresponding upper clamping force $F_{1}$ can be calculated from Equation (15).

With the increase of $F_{2}$, the deflections of the stacked plates can be divided into four stages listed below:

1. Stage1: When the lower clamping force $F_{2}=0$, the one-sided clamping force is adopted. The upper clamping force $F_{1}$ is the largest among all groups of bidirectional clamping forces at this stage. The deflection of the lower plate is induced by the drilling thrust force $F_{d}$, while the upper plate deforms downward due to the upper clamping force until the interlayer gap is eliminated. At this stage, the deflection of the upper plate $\omega_{u}$ is more significant than the lower plate $\omega_{d}$, and their difference is equal to the initial gap $\mu_{0}$.
2. Stage2: As $F_{2}$ increases, the deflections of both plates decrease until the effect of $F_{2}$ counteracts the effect of the drilling force on the lower plate. At the end of stage 2, the deflection of the upper plate $\omega_{u}$ is equal to the initial gap $\mu_{0}$ while the deflection of the lower plate $\omega_{d}$ is zero.
3. Stage3: When $F_{2}$ is greater than the drilling thrust force $F_{d}$, the lower plate begins to deform upward, and the downward deformation of the upper plate decreases accordingly. In this stage, the sum of the deflections of the two stacked plates is always equal to the initial gap $\mu_{0}$. When the deflections of the upper and lower plates are the same ($\omega_{u}=\omega_{d}=\mu_{0}/2$), the non-uniformity of the deformation is the smallest. The bidirectional clamping forces at this time can be considered as optimal.
4. Stage4: As $F_{2}$ continues to increase, $F_{1}$ finally reaches zero. At this moment, the deflection of the upper plate $\omega_{u}$ is zero, the lower clamping force $F_{2}$ is the maximum, and the deflection of the lower plate $\omega_{d}$ is equal to the initial gap.

According to the above analysis, when the deflections of the upper and lower plates are equal to half of the initial gap ($\omega_{u}=\omega_{d}=\mu_{0}/2$), the optimal bidirectional clamping forces can be obtained, the calculation results are $F_{1}=62.9\;N$ and $F_{2}=192.3\;N$.

## 3. Numerical Study

### 3.1. Finite Element Modeling of Drilling Stacked Plates

In order to verify the feasibility and correctness of the interlayer gap model and the optimal bidirectional clamping forces obtained from theoretical analysis. A 3D finite element model of drilling double-layer stacked plates under bidirectional clamping forces was established using ABAQUS 6.14. The FE modeling is based on the research of Tian et al. [12]. The static, general procedure of simulation was performed, and the modeling steps are as follows:

(1) Geometric model

**Figure 6:**
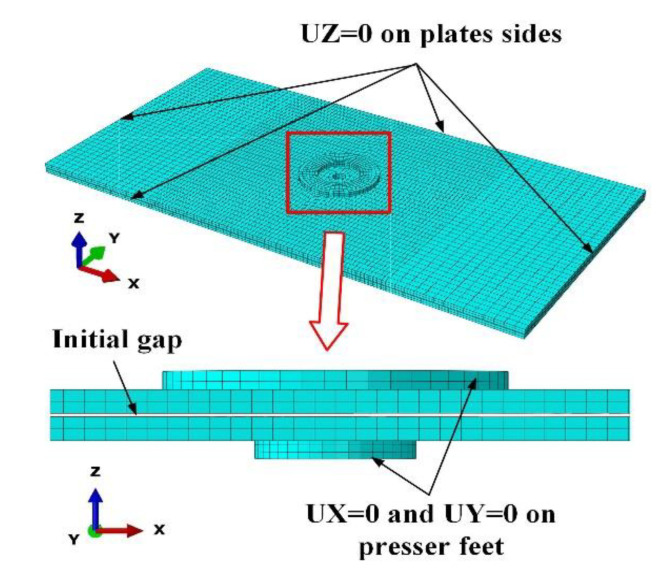
The finite element (FE) model of the stack drilling with bidirectional clamping forces.

The geometric model shown in Figure 6 consists of four parts: two aluminum alloy plates, the upper presser foot and the lower presser foot. The drill is replaced with a concentrated force at the drilling center. The stacked plates are built as deformable bodies, while the presser feet are defined as rigid bodies. In accordance with the theoretical model, the size of the stacked plates is 200×100×2 mm. The upper presser foot is a ring with an inner radius of 15 mm and an outer radius of 8 mm. Similarly, the inner and outer radius of the lower presser foot is 7 and 5 mm, respectively. The initial gap is set to 0.35 mm [27].

(2) Material definition

The material definition of the FE model is the same as the theoretical model. The material of the stacked plates is set as aluminum alloy 2024-T3, and the presser feet are defined as stainless steel. The material properties are shown in Table 1.

(3) Mesh selection

As shown in Figure 6, the hexahedral element C3D8R reduced integration 8-node solid continuum element is used to generate the meshing of the components. Moreover, encrypted meshing is performed on areas near the drilling center on the plates. The meshing size of the plates is 1.6–2.5 mm in different areas, while the meshing size of the presser feet is 2.0 mm.

(4) Load and boundary condition

The drilling thrust force is set to 130 N during the simulation. There are contacts between the upper and lower plates and between the presser feet and plates in the FE model. The contact type is set as surface to surface contact with a friction coefficient of 0.2 [12]. The clamping forces are applied on the presser feet while the drilling thrust force is loaded at the drilling center of the lower plate. The four sides of the plates are constrained in the Z-direction to satisfy the requirement of simply supporting. The presser feet restrict all degrees of freedom except the Z-direction so that the clamping forces can be applied.

With the established FE model, the interlayer gap and the deflections of the upper and lower plates under different clamping forces can be obtained.

### 3.2. Simulation Results and Discussion

According to Equation (15), six groups of bidirectional clamping forces are selected for simulation. As shown in Table 4, the lower clamping force $F_{2}$ increases from 0 to 251.2 N from group 1 to group 6, while the upper clamping force $F_{1}$ decreases from 268.3 N to 0. Besides, it should be noticed that group 1 is one-sided clamping with only upper clamping force, group 5 has the theoretical optimal bidirectional clamping forces, and group 6 is one-sided clamping with only lower clamping force.

**Figure 7:**
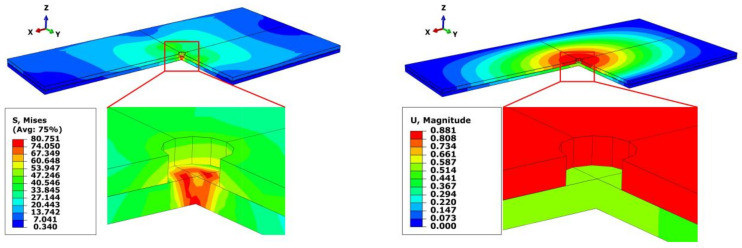
Stress and strain cloud diagrams of group 1 (one-sided clamping force).

**Figure 8:**
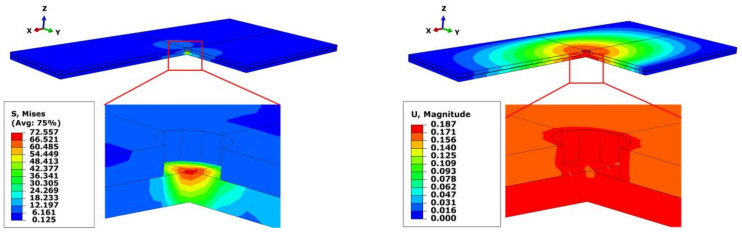
Stress and strain cloud diagrams of group 5 (optimal bidirectional clamping forces).

Taking group 1 and group 5 as examples, the cloud diagrams of stress and strain are illustrated in Figure 7 and Figure 8, respectively. As can be seen, the maximum stress and strain of group 5 are less than that of group 1. Meanwhile, the difference of the deflections between the upper and lower plates is 0.05 mm in group 5, which is far less than 0.3 mm in group 1. The results sufficiently prove the superiority of the bidirectional clamping method.

**Figure 9:**
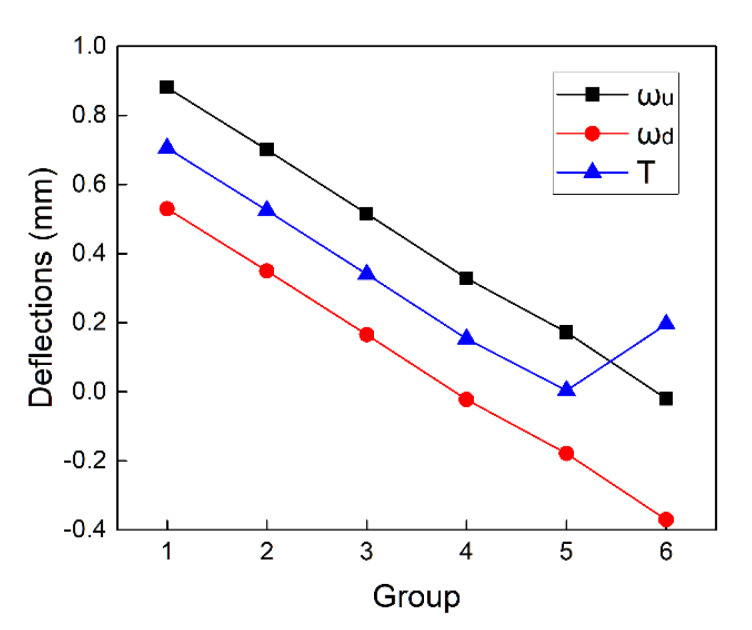
The deflection of stacked plates with different groups of bidirectional clamping forces.

The simulation results of different groups of bidirectional clamping forces are shown in Table 5. As can be seen, the interlayer gap of all groups is less than 0.0015 mm, which means the interlayer gaps are basically eliminated. Therefore, it can be considered that the bidirectional clamping forces satisfying Equation (15) can control the interlayer gap and limit the interlayer burr formation. The deflections of the upper and lower plates and the index T in Equation (16) of six groups are illustrated in Figure 9.

It can be seen that from group 1 to group 4 ($F_{2}$ increases from 0 to 150 N), the deflections of the upper and lower plates both decrease. Then in group 5, $\omega_{u}=0.1772\;\mathrm{mm}$, $\left|\omega_{d}\right|=0.1784\;\mathrm{mm}$, the deflections of upper and lower plates are very close to the half of the initial gap ($\mu_{0}/2=0.175\;\mathrm{mm}$), the deflection evaluation index T is the smallest in this group. In group 6, the lower clamping force reaches the maximum value, while the upper clamping force becomes zero. The evaluation index T increases in the last group, which represents the increase of the non-uniformity of deformations. In summary, the deflections of the stacked plates in group 5 are the smallest and the most uniform, which indicates that the calculation result of the optimal clamping forces in the theoretical analysis is correct and valid.

## 4. Experiments

### 4.1. Experimental Setups

**Figure 10:**
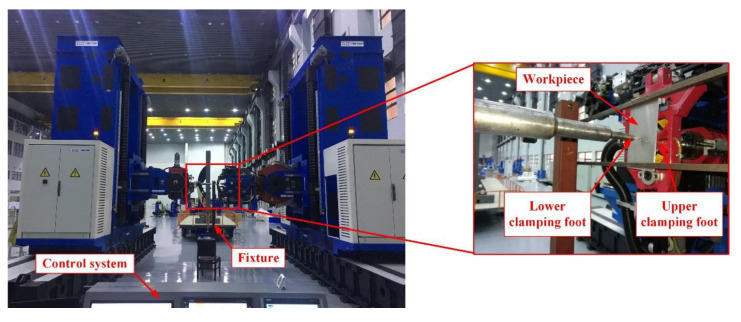
The drilling experiment platform based on the automatic drilling and riveting system.

In order to verify the effect of the optimized bidirectional clamping forces on burr formation in the practical machining environment, drilling experiments were carried out based on the automatic drilling and riveting system. The experiment layout of the stack drilling with bidirectional clamping forces is shown in Figure 10. Some 2024-T3 aluminum alloy plates of 200×100×2 mm were used for the double-layered stack drilling experiments. The material properties are listed in Table 1. The cutting tool used is a ϕ5.1 mm cemented carbide drill, with a 120° point angle and a 15° spiral angle. The upper and lower clamping is realized by the pneumatic presser feet installed on the end effectors of the automatic drilling and riveting system. The drilling thrust force and clamping forces were measured by a KISTLER9257B dynamometer (Kistler Group, Winterthur, Switzerland).

The experiments consist of two parts, and the details are described in the next section. In all of the experiments, the same processing parameters of 6000 r/min spindle speed and 600 mm/min feed speed were utilized to obtain the same drilling thrust force 130 N. After experiments, the Zeus Axio CSM 700 confocal microscope (Carl Zeiss Microscopy GmbH, Jena, Germany) was applied to observe and measure the interlayer burrs.

### 4.2. Experimental Results and Discussion

**Figure 11:**
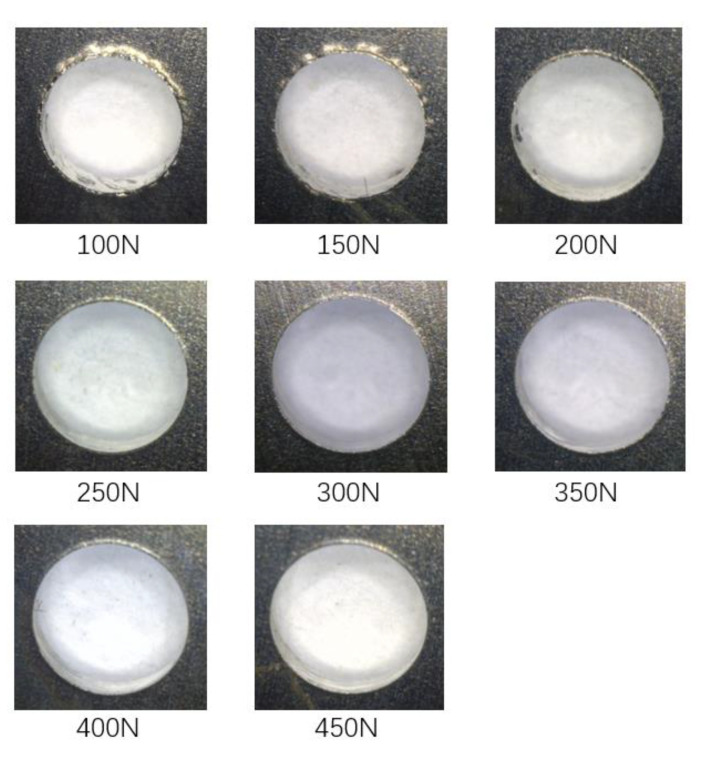
The burrs at the exit of the upper plates under different upper clamping forces.

**Figure 12:**
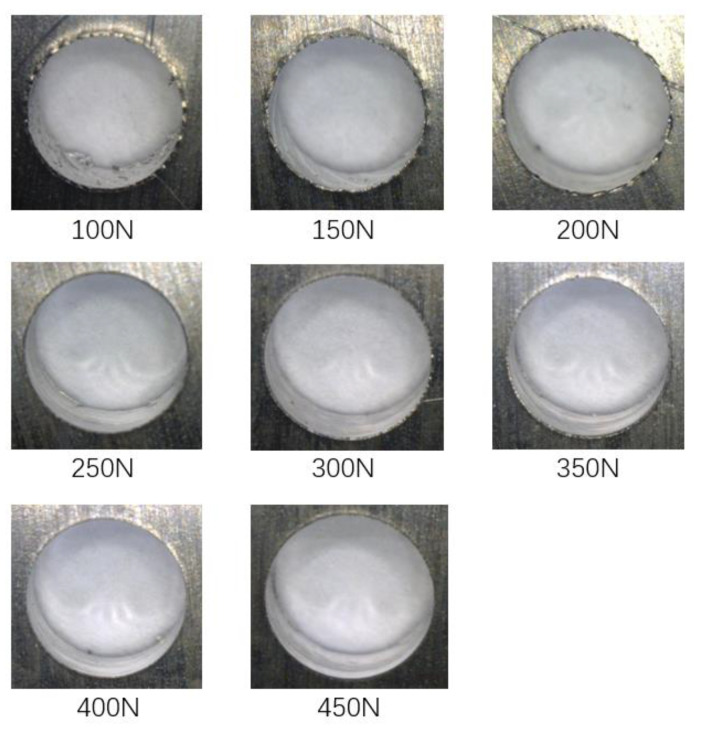
The burrs at the entrance of the lower plates under different upper clamping forces.

In part 1, the lower clamping force is fixed at 50 N, while the upper clamping force varies from 100 N to 450 N with an interval of 50 N. Three holes were drilled under each force level. The primary purpose of part 1 is to analyze the variation of the interlayer burr under different clamping forces and study the inhibition effect of bidirectional clamping forces on the interlayer burr. The burrs at the exit of the upper plate and the entrance of the lower plate are illustrated in Figure 11 and Figure 12. It can be seen that when the upper clamping force is less than 250 N, the interlayer burrs turn smaller with the increase of the clamping force. After the upper clamping force exceeds 250 N, the burr morphologies at the hole area become stable, and there is basically no interlayer burr generated.

**Figure 13:**
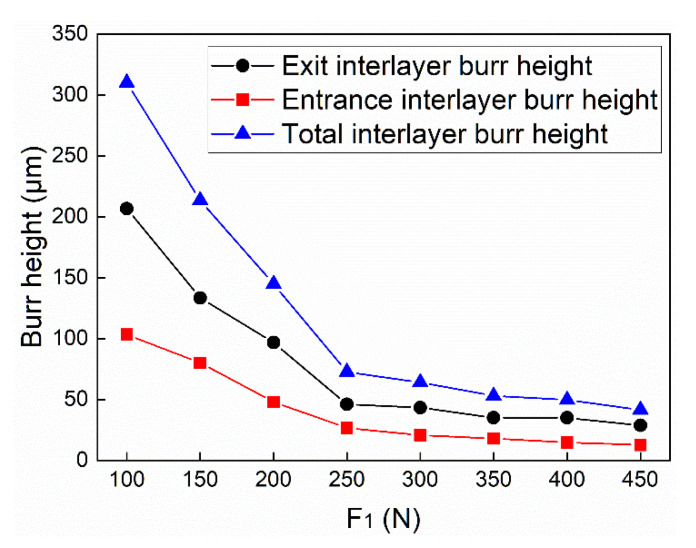
The burr height under different upper clamping forces.

The interlayer burr heights were measured and averaged under different upper clamping forces. The results are exhibited in Figure 13. The measured results further prove the correctness of the observation. The burr height decreases significantly with the increase of the upper clamping force. When the upper clamping force reaches 250 N, the trend slows down. The burr height is finally stabilized at about 0.05 mm. It proves that bidirectional clamping forces can effectively inhibit the interlayer burr formation.

The formation of interlayer burrs is affected by factors such as the clamping forces, the cutting parameters, tool parameters, material parameters, pre-connection conditions and stiffness of metal plates, etc. Additionally, there are two forces that directly affect the size of interlayer burrs. One is the axial force from the drilling bit, which deforms the lower plate and forms the initial gap. The other is the plastic flow stress produced by the cutting and extruding of the workpiece. The flow stress squeezes the materials into the initial gap between stacks, thereby forming the interlayer burrs. Under one-sided clamping, the lower plate is not supported by the presser foot and the plastic flow force will squeeze the lower plate away. However, with bidirectional clamping, the lower presser foot can significantly restrain the extrusion effect of plastic flow stress on the lower plate, thus effectively reducing the interlayer gap and interlayer burr.

**Figure 14:**
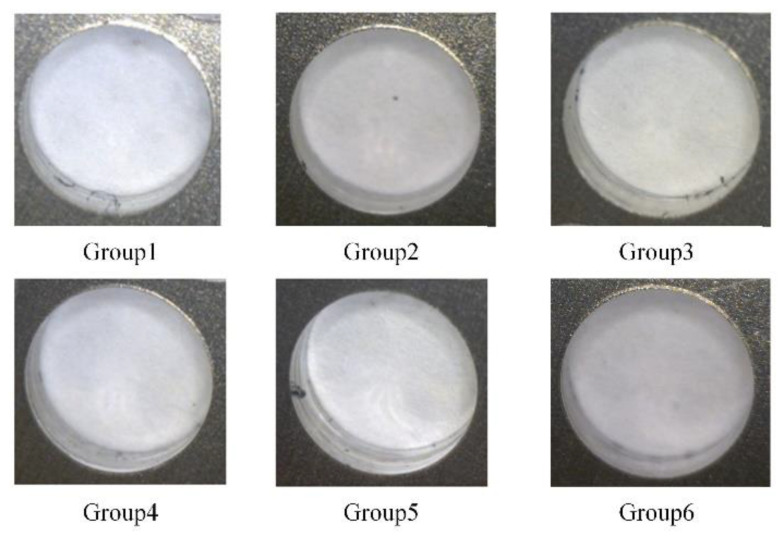
The burrs at the exit of the upper plates with six groups of the bidirectional clamping forces in Table 4.

**Figure 15:**
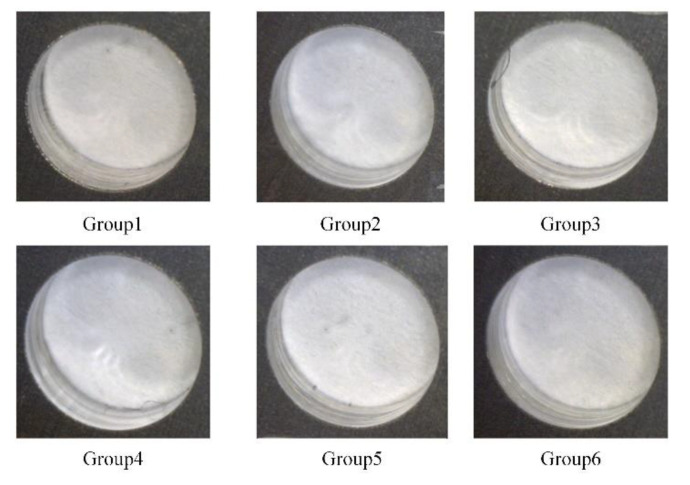
The burrs at the entrance of lower plates with six groups of the bidirectional clamping forces in Table 4.

Moreover, in part 2 of the experiments, the six groups of the bidirectional clamping forces in Table 4 of the simulation are selected to perform drilling experiments. Since the bidirectional clamping forces in Table 4 satisfy the optimized relationship formula Equation (15) in theoretical analysis, therefore, if the interlayer burrs are eliminated with the optimized bidirectional forces, the theoretical analysis and the simulation results can be validated. Similarly, three holes were drilled with each group of clamping forces. The observed burr morphologies are illustrated in Figure 14 and Figure 15.

**Figure 16:**
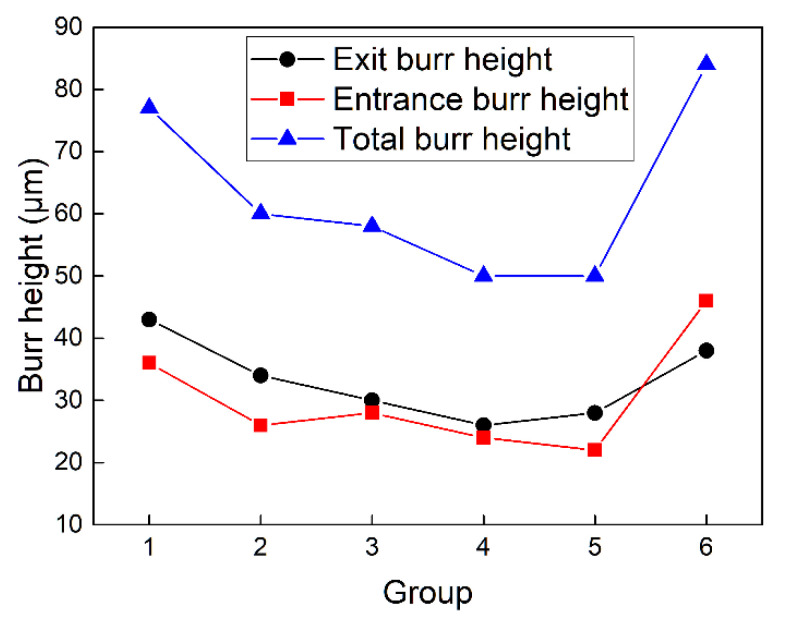
The burr heights with six groups of the bidirectional clamping forces in Table 4.

It can be seen that in all six groups, there is no visible burr at the exit of the upper plates and entrance of the lower plates, the inner surface of the hole is smooth, and the burr-free drilling is achieved. This phenomenon indicates that the interlayer gap is inhibited and the interlayer burr is eliminated. Furthermore, the average burr heights of the six groups of bidirectional clamping forces are measured and presented in Figure 16.

It can be seen from Figure 16 that in all six groups, the total interlayer burr height is less than 100 μm, which indicates that the optimized bidirectional clamping forces determined by the theoretical analysis are sufficient, and the aim of burr-free drilling can be achieved. Moreover, it should be noticed that the burr heights in different groups of bidirectional clamping forces have a rather significant difference. The total interlayer burrs in group 1 and 6 (around 80 μm) are more extensive than the other groups with bidirectional clamping forces (around 55 μm). The main reason is in group 1 and 6, one-sided clamping is adopted, so the bending of the plate without presser foot cannot be restricted. Specifically, in group 1, the bending of the lower plate causes a larger burr at the entrance of the lower plate. In group 6, when the tool is drilled into the lower plate, the upper plate will produce relative movement due to the lack of clamping force. This also results in a larger burr between the stacked plates. Meanwhile, the tool wear and measurement error will also affect the measurement accuracy of burr height. The experimental results of part 2 prove that the optimized bidirectional clamping forces satisfying Equation (15) can significantly inhibit the interlayer gap and eliminate the interlayer burrs.

In summary, the experimental results of two parts verify the correctness, feasibility and validity of the optimization results of the bidirectional clamping forces in theoretical analysis and FE model.

## 5. Conclusions

In aircraft assembly, single-shot drilling of the aluminum alloy stacked plates is widely applied to improve the assembly precision and efficiency. A significant way to eliminate interlayer burr is to control the interlayer gap with preloading clamping force. The application of the dual-machine-based automatic drilling and riveting system has promoted the research requirement of burr-free drilling with bidirectional clamping forces. In this paper, through theoretical, simulation and experimental research, the following conclusions have been drawn:

(1) Preloading clamping force can effectively reduce the interlayer gap, thereby eliminate the formation of interlayer burr, and the effect of bidirectional clamping is better than that of one-sided clamping.
(2) Based on the theory of plates and shells, the interlayer gap theoretical model is established. The relationship between the upper and lower clamping forces is derived to ensure the interlayer gap is zero. Under the processing conditions in this paper, their relationship formula is $F_{1}+1.068F_{2}=268.3\;N$.
(3) When the interlayer gap is zero, four different stages of the stacked plates deflections are revealed based on the interlayer gap theoretical model. Moreover, when the deflection of the upper and lower plates is equal to half of the initial gap, the total deformation of the stacked plates is the smallest, and neither plate will have excessive deflection.

This paper provides a theoretical basis for the practical application of bidirectional clamping in dual-machine-based drilling. Furthermore, the research results can be transferred to the drilling of composite materials. The bidirectional clamping can inhibit the delamination of the composite materials to a certain extent, and that will be the focus of our further research.

## Figures and Tables

**Table 1:** Material properties of different components of the drilling system.

Component	Material	Elastic Modulus/GPa	Poisson’s Ratio
Plate	2024-T3 aluminum alloy	72	0.34
Presser foot	Stainless steel	200	0.3
Drill	Cemented carbide	-	0.3

**Table 2:** Geometric dimensions of the plates and the bidirectional presser feet (unit: mm).

Component	Parameter	Value
Plate	Length (a)	200
	Width (b)	100
	Thickness (h)	2
Upper presser foot	Inner radius ($R_{u1}$)	8
	Outer radius ($R_{u2}$)	15
Lower presser foot	Inner radius ($R_{d1}$)	5
	Outer radius ($R_{d2}$)	7

**Table 3:** The deflection of the plate at different radius under each unit force (unit: 10^−6^ m/N).

r/(mm)	ω(O→r)	$\omega \left(R_{u}\to r\right)$	$\omega \left(R_{d}\to r\right)$
0	3.0594	2.7865	2.9770
2	3.0518	2.7828	2.9711
4	3.0294	2.7719	2.9537
6	2.9939	2.7535	2.9255
8	2.9475	2.7276	2.8872
10	2.8927	2.6941	2.8404
12	2.8318	2.6536	2.7866
14	2.7673	2.6064	2.7274
20	2.5288	2.5603	2.4350
30	2.1615	2.1843	2.0972

**Table 4:** Different bidirectional clamping forces groups for simulation (unit: N).

Group	$\mathrm{Upper}\;\mathrm{Clamping}\;\mathrm{Force}\;(F_{1})$	$\mathrm{Lower}\;\mathrm{Clamping}\;\mathrm{Force}\;(F_{2})$	Remark
1	268.3	0	One-sided clamping
2	214.9	50	-
3	161.5	100	-
4	108.1	150	-
5	62.9	192.3	Optimal bidirectional clamping
6	0	251.2	One-sided clamping

**Table 5:** Simulation results of different groups of bidirectional clamping forces (unit: mm).

Group	$\mathrm{Interlayer}\;\mathrm{Gap}\;(\mu_{0})$	$\mathrm{Upper}\;\mathrm{Plate}\;\mathrm{Deflection}\;(\omega_{u})$	$\mathrm{Lower}\;\mathrm{Plate}\;\mathrm{Deflection}\;(\omega_{d})$	Deflection Index T in Equation (16)
1	0.0008	0.8806	0.5298	0.7052
2	0.0008	0.7004	0.3496	0.5250
3	−0.0001	0.5151	0.1652	0.3402
4	−0.0005	0.3281	−0.0224	0.1529
5	0.0005	0.1722	−0.1784	0.0031
6	0.0013	−0.0205	−0.3706	0.1955

## Nomenclature

$F_{d}$	The drilling thrust force
$P_{u}$ and $P_{d}$	The pressures applied on the clamping areas of upper side and lower side
$R_{u1}$, $R_{u2}$, $R_{d1}$, and $R_{d2}$	The inner radius and outer radius of the upper and lower presser feet
$F_{1}$	The upper clamping force at circle with radius $R_{u}$
$F_{2}$	The lower clamping force at circle with radius $R_{d}$
$\mu_{0}$	The initial gap
μ	The interlayer gap
a	The length of the sheet;
b	The width of the sheet
h	The thickness of the sheet
ω(O→r)	The deflection at the circle of radius r caused by unit force at drilling center
$\omega \left(R_{u}\to r\right)$	The deflection at the circle of radius r caused by unit force at upper clamping area
$\omega \left(R_{d}\to r\right)$	The deflection at the circle of radius r caused by unit force at lower clamping area
$\omega_{1}\left(r\right)$	The deflection at the circle of radius r of upper plate
$\omega_{2d}\left(r\right)$	The deflection of the plate caused by the thrust force
$\omega_{2c}\left(r\right)$	The deflection of the lower plate caused by the lower clamping force
∆ω(r)	The interlayer gap
$\omega_{u}\left(r\right)$	The deformation of the upper plate
$\omega_{d}\left(r\right)$	The deformation of the lower plate
ω	The deflection of the rectangular thin plate
P(x,y)	A point on the plate
$P_{0}\left(x_{0},y_{0}\right)$	The position of the drilling thrust force applied at the lower plate
$P_{1}\left(x_{1},y_{1}\right)$ and $P_{2}\left(x_{2},y_{2}\right)$	The positions of the upper and lower clamping forces applied on the plates
